# Plasma Small Extracellular Vesicles Derived miR-21-5p and miR-92a-3p as Potential Biomarkers for Hepatocellular Carcinoma Screening

**DOI:** 10.3389/fgene.2020.00712

**Published:** 2020-07-23

**Authors:** Andrei Sorop, Razvan Iacob, Speranta Iacob, Diana Constantinescu, Leona Chitoiu, Tudor Emanuel Fertig, Anca Dinischiotu, Mihaela Chivu-Economescu, Nicolae Bacalbasa, Lorand Savu, Liliana Gheorghe, Simona Dima, Irinel Popescu

**Affiliations:** ^1^Center of Excellence in Translational Medicine, Fundeni Clinical Institute, Bucharest, Romania; ^2^Faculty of Biology, University of Bucharest, Bucharest, Romania; ^3^“Carol Davila” University of Medicine and Pharmacy, Bucharest, Romania; ^4^Digestive Diseases and Liver Transplantation Center, Fundeni Clinical Institute, Bucharest, Romania; ^5^Ultrastructural Pathology Laboratory, Victor Babeş National Institute, Bucharest, Romania; ^6^Department of Cellular and Molecular Pathology, Stefan S. Nicolau Institute of Virology, Bucharest, Romania; ^7^“Titu Maiorescu” University of Medicine and Pharmacy, Bucharest, Romania

**Keywords:** exosomes, microRNA, serum biomarkers, hepatocellular carcinoma, screening

## Abstract

**Introduction:**

Liquid biopsy using circulating microvesicles and exosomes is emerging as a new diagnostic tool that could improve hepatocellular carcinoma (HCC) early diagnosis and screening protocols. Our study aimed to investigate the utility of plasma exosomal miR-21-5p and miR-92-3p for HCC diagnosis during screening protocols.

**Methods:**

The study group included 106 subjects: 48 patients diagnosed with HCC during screening, who underwent a potentially curative treatment (surgical resection or liver transplantation), 38 patients with liver cirrhosis (LC) on the waiting list for liver transplantation, and 20 healthy volunteers. The exosomes were isolated by precipitation with a reagent based on polyethylene glycol and were characterized based on morphological aspects (i.e., diameter); molecular weight; CD63, CD9, and CD81 protein markers; and exosomal miR-21-5p and miR-92a-3p expression levels.

**Results:**

We first demonstrate that the exosome population isolated with the commercially available Total Exosome Isolation kit respects the same size ranging, morphological, and protein expression aspects compared to the traditional ultracentrifugation technique. The analysis of the expression profile indicates that miR-21-5p was upregulated (*p* = 0.017), and miR-92a-3p was downregulated (*p* = 0.0005) in plasma-derived exosomes from HCC subjects, independently from the patient’s characteristics. AUROC for HCC diagnosis based on AFP (alpha-fetoprotein) was 0.72. By integrating AFP and the relative expression of exosomal miR-21-5p and miR-92a-3p in a logistic regression equation for HCC diagnosis, the combined AUROC of the new exosomal miR HCC score was 0.85—significantly better than serum AFP alone (*p* = 0.0007).

**Conclusion:**

Together with serum AFP, plasma exosomal miR-21-5p and miR-92a-3p could be used as potential biomarkers for HCC diagnosis in patients with LC subjected to screening and surveillance.

## Introduction

Hepatocellular carcinoma (HCC) is the fifth most common cancer worldwide and the second most frequent cause of cancer-related death globally. It is closely linked to the presence of an underlying chronic liver disease and has a high clinical and biological heterogeneity (Laurent-Puig et al., 2001; Akinyemiju et al., 2017). Early detection of HCC is critical for a curative treatment and for improving long-term outcomes of patients. HCC detection at an early stage is challenging as the disease usually progresses asymptomatically. Thus, screening programs are used for cancer detection in patients at risk for tumor development and usually include ultrasound (US) with or without serum biomarkers, such as alpha-fetoprotein (AFP) (Galle et al., 2018; Heimbach et al., 2018). Pooled sensitivity of US for any HCC stage in a meta-analysis of 32 studies (Tzartzeva et al., 2018) was good (84%, 95% CI 76–92), but it decreased to a modest value (47%, 95% CI 33–61) for the detection of early stage HCC. Serum AFP levels also exhibit a low sensitivity for early HCC diagnosis, ranging from 20 to 65% (El-Serag and Davila, 2011; Tian et al., 2017).

Biomarker quantification using blood samples is less expensive than radiological imaging and could potentially detect early stage HCC even before it could be identified by conventional imaging (Johnson et al., 2014). The use of other biomarkers, such as AFP isoforms or des-gamma-carboxy prothrombin (DCP), has been proposed to improve the accuracy of AFP in the early detection of HCC and subsequent therapeutic response monitoring (Lim et al., 2016; Cerban et al., 2018; Li and Chen, 2019). The GALAD scoring algorithm, comprising gender, age, AFP-L3, AFP, and DCP, has very good performance for HCC detection in large validation studies, including patients of diverse liver disease etiologies, even for detecting early stage HCC (BCLC 0/A) (Berhane et al., 2016; Best et al., 2019). Advances in genomics and proteomics platforms have resulted in the identification of numerous other novel biomarkers and have improved the diagnostic accuracy of HCC. The study of these biomarkers has contributed to the clarification of mechanisms driving hepatocarcinogenesis, facilitating personalized diagnosis and treatment strategies.

Over the past decade, research on small extracellular vesicles (EVs)–exosomes has made significant advances, highlighting their important role as microRNA (miRNA) carriers, disease biomarkers, and potential therapeutic targets (Ailawadi et al., 2015; Yanez-Mo et al., 2015). MiRNAs are highly conserved small non-coding RNAs, that influence the expression of almost 30% of human protein-coding genes at the posttranscriptional and the translational levels (Fu et al., 2012). Exosomes are a specific population of EVs defined by their diameter, between 40 and 150 nm (Malm et al., 2016).

Exosomes are involved in HCC progression by regulating proliferation, angiogenesis, and invasion of tumor cells. Exosomes may also regulate HCC hypoxia stress and drug resistance. Based on these observations, exosomes are emerging as novel biomarkers for liver diseases including HCC (Cai et al., 2017). Exosomes carry a wide range of components, such as miRNAs, mRNAs, transcription factors, proteins, and lipids. This content is highly variable and depends on cellular origin, thus performing powerful and transmissible functions on recipient cells (Melo et al., 2015; Milane et al., 2015). Many studies have shown that circulating miRNAs derived from exosome species are involved in the development of HCC and can be considered as possible diagnostic or prognostic markers (Xu et al., 2011).

The aim of the present study was to investigate the clinical utility of plasma exosomal miR-21-5p and miR-92a-3p quantification for HCC diagnosis as potential new biomarkers for HCC screening programs.

## Materials and Methods

### Patients and Sample Collection

A total number of 106 subjects were included in this study after written informed consent according to the Guidelines of the Fundeni Clinical Institute Ethics Committee (30884/22.10.2014 and 29435/12.09.2016), which are subject to the Helsinki Declaration on ethical principles for medical research involving human subjects. The patients were divided into three groups: 48 patients diagnosed with HCC during the screening program, who underwent a potentially curative treatment at the Digestive Diseases and Liver Transplantation Center, Fundeni Clinical Institute (all cases had a confirmed HCC diagnosis by pathological examination following the therapeutic procedure); 38 patients included on the waiting list for liver transplantation for liver cirrhosis (LC) and, thus, during the screening program for HCC; and as a control group (C) 20 healthy volunteers. Peripheral blood samples from all the subjects were collected in 5-mL vacutainer (Becton Dickinson) tubes spray-coated with EDTA as anticoagulant and centrifuged at 4200 × rpm for 10 min at room temperature (RT) to separate the plasma fraction. The plasma was transferred to fresh tubes and stored at −80°C until further analysis.

### Plasma Exosome Isolation

Exosome isolation was performed by two well-known techniques. The Total Exosome Isolation (TEIp) kit is based on polyethylene glycol precipitation (PEG) and ultracentrifugation (UC). The TEIp kit (Invitrogen, Life Technologies) was used to isolate exosomes (small EVs) from the plasma following the manufacturer’s protocol with minor modifications (Rider et al., 2016; Moon et al., 2019). First, plasma samples were thawed at 37°C for 1 min in a water bath. Exosomes were isolated from approximately 2 mL of cryopreserved plasma by differential centrifugation at 3000 × *g* for 20 min and at 15,000 × *g* for 20 min at 4°C to completely remove the cellular components. Briefly, 0.5 volumes of 1× PBS and 0.05 volumes of Proteinase K were added to the clarified plasma by mixing and incubated at 37°C for 10 min. To the mixed solution, 0.2 volumes of total exosome isolation reagent was added and incubated on ice for 1 h, followed by centrifugation at 10,000 × *g* for 10 min at 4°C. Finally, the exosome pellets were resuspended in the appropriate volume for characterization and RNA isolation.

One milliliter of plasma for characterization methods was ultracentrifuged according to the method previously described (Thery et al., 2006) with some protocol modifications. Briefly, plasma was centrifuged at 3000 × *g*, 30 min, and 4°C to remove cell debris. Then, the supernatants were collected in ultracentrifuge tubes, and 1× PBS was added to up to two thirds of the tubes and centrifuged at 29,500 × *g* for 45 min, 4°C to remove large particles. Next, we filtrated the supernatants with a 0.22-μm filter, and the exosomes were pelleted with ultracentrifugation at 120,000 × *g* for 2 h, 4°C in an SW-40-Ti swinging-bucket rotor, Optima XPN-100 ultracentrifuge instrument (Beckman Coulter, Brea, CA, United States). The exosome pellets were resuspended in appropriate volumes for further experiments.

The characterization of the patient’s plasma-derived exosomes was made through the following methods: nanoparticle tracking analysis, negative stain, transmission electron cryomicroscopy (cryoTEM), and western blotting (Tang et al., 2017).

### Nanoparticle Tracking Analysis

The size distribution of small EVs was determined using a Delsa Nano Analyzer (DelsaNano, Beckman Coulter, Brea, CA, United States). This instrument utilizes photon correlation spectroscopy (PCS) and electrophoretic light scattering techniques to determine the size distribution and zeta potential of exosomes. The capture data and analysis settings for intensity distribution were performed manually according to the manufacturer’s instructions.

### Negative Stain

Screening of specimens by negative stain in TEM represented a quick method to analyze the distribution of particles and to select an optimal dilution for cryoTEM. Copper grids (100 mesh) coated with formvar and carbon films were glow discharged to increase the binding of particles to the support film. A volume of 5 μl of sample was incubated for 2 min on grids at RT. Excess sample was removed by blotting. The grids were stained with three successive drops of 2% uranyl acetate with excess stain again removed by blotting. Image acquisition was done at RT using a 200 kV Talos F200C TEM (Thermo Fisher Scientific) under similar imaging conditions as for cryoTEM.

### Transmission Electron Cryomicroscopy

Samples were embedded in a thin layer of vitreous ice by rapid plunging in liquid nitrogen (LN_2_)-cooled ethane, using a Leica grid plunging system (Leica EM GP, Leica Microsystems, Wetzlar, Germany). Briefly, copper grids (Quantifoil R2/2, Quantifoil Micro Tools, Großlöbichau, Germany) were glow discharged and then incubated for 2 min at 90% humidity with a 5-μl droplet of isolated EVs and finally blotted for 5 s before plunging. The grids were then transferred under liquid LN_2_ to the cold stage of a 200 kV Talos F200C TEM (Thermo Fisher Scientific) equipped with a 4 k × 4 k Ceta camera. Specimens were examined in the low-dose mode, at maximum 8 electrons per Å^2^ and 8–12 μm under focus. Images acquired at 28,000×, 36,000×, and 45,000× nominal magnifications gave a final object sampling of 5.2, 4.1, and 3.2 Å, respectively.

### Western Blotting Analysis

The exosome concentration was determined indirectly by quantifying the protein concentration in exosome lysates. Isolated exosomal pellets from the plasma were washed in phosphate-buffered saline, mixed with RIPA lysis buffer containing Complete O^TM^, Mini, EDTA-free Protease Inhibitor Cocktail (Roche Applied Science). The protein concentration of exosome lysates was determined using BCA protein assay (Pierce, Thermo Fisher Scientific). The absorbance was read at 562 nm using a TriStar^2^ S LB 942 plate reader (Berthold). To ascertain the isolation of the exosomes from plasma samples, western blotting analysis was established for the cluster of differentiation 63 (CD63), CD9, and CD81. Whole protein extracts (60 μg) were electrophoretically separated by SDS-PAGE and transferred onto polyvinylidene fluoride (PVDF) membranes that were subsequently blocked in tris-buffer saline (TBS)-0.5% Tween 20 with 2% bovine serum albumin and then incubated with the primary antibodies against proteins of interest at 4°C overnight. The following monoclonal primary antibodies were used: mouse anti-CD63 (ID: 10628D, clone Ts63) (1:250), mouse anti-CD9 (ID: 10626D, clone Ts9) (1:250), and mouse anti-CD81 (MA5-13548, clone 1.3.3.22) (1:250), all from Invitrogen (Life Technologies). Mouse monoclonal anti-β-actin (clone Ac-74) (Sigma-Aldrich) (1:2000) was used as a control. Membranes were then washed with TBS-T (0.5% Tween-20) and incubated 2 h with secondary antibodies anti-mouse conjugated with HRP (RD Systems) (1:2000). Signals were developed using ECL HRP chemiluminescent substrate (Invitrogen) and captured using MicroChemi 4.2 system (Bio Imaging Systems).

### Isolation of Exosomal RNA

The RNA was extracted from exosomes that have been isolated with a TEIp kit. Isolation of total RNA from exosomes was performed by the standard method with TRIzol (Invitrogen, Thermo Fisher Scientific) according to manufacturer specifications with several modifications: 1 mL of TRIzol was added to the exosome samples and incubated overnight at −20°C. After incubation, 300 μl of chloroform was added and centrifuged at 12,000 × *g* for 15 min at 4°C to separate a clear upper aqueous layer containing total RNA that precipitates with 1 volume of isopropanol 100% and glycogen (20 mg/mL) as a carrier. Samples were incubated for 1 h on ice to get a maximum recovery of the amount of RNA species from exosomes (including miRNAs). Exosomal RNA samples were centrifuged at 15,000 × *g*, and the pellet was washed twice in 1 mL of 75% ethanol. Subsequently, the pellet was air-dried and eluted in 15 μl RNase- and DNase-free water. The quantity of the RNA was determined by the concentration and purity (A260/A280 and A260/A230) assessed by NanoDrop ND1000 (NanoDrop Technologies, Waltham, MA, United States). Quality and size distribution pattern of exosomal RNA was analyzed using chip-based capillary electrophoresis Agilent Bioanalyzer 2100 with a Small RNA Chip and Pico RNA Chip (Agilent Technologies, Santa Clara, CA, United States), according to manufacturer protocol.

### Assessment of Plasma Exosomal miRNA Levels Using Quantitative Real-Time PCR

MiRNA profiling was performed for the exosome samples isolated by the TEIp kit, and the expression levels were quantified by real-time quantitative PCR (RT-qPCR). TaqMan probes for miR-21-5p (ID: 000397) and miR-92a-3p (ID: 000431) (Applied Biosystems, Thermo Fisher Scientific) were used for quantitative real-time PCR (qRT-PCR). A TaqMan MicroRNA Reverse Transcription Kit (Applied Biosystems, Thermo Fisher Scientific) was used to synthesize cDNA by incubation as follows: 4°C for 5 min, 16°C for 30 min, 42°C for 30 min, and 85°C for 5 min. The amplification step was performed by TaqMan Universal PCR Master Mix, No AmpErase UNG (Applied Biosystems, Thermo Fisher Scientific) with the following thermocycler protocol: 95°C for 10 min + (95°C for 15 s; 60°C for 60 s) for 40 cycles. The ABI PRISM 7300 Sequence Detection System (Applied Biosystems, Thermo Fisher Scientific) was used to analyze miR-21-5p, miR-92a-3p relative expression normalized to miR-16-5p (ID:000391) (Davoren et al., 2008; Matsumura et al., 2015).

The expression level of the three miRNAs are related as fold changes 2^–△△^^Ct^ (see section “Materials and Methods” in Supplementary Data S1) above to the mean expression level determined by assessing the healthy controls (*n* = 20), thus providing a quantitative parameter to define the exosomal expression of miRNAs in HCC and LC samples, respectively, that was used in subsequent statistical analyses.

### Statistical Analysis

Categorical variables were expressed as percentages of respective populations and compared between study groups using the Chi-square test. Continuous variables, including the expression levels of the analyzed exosomal miRNAs, were compared between study groups using the Mann–Whitney *U* test or Student’s *t-*test when appropriate. Logistic regression was used to generate a multiparametric predictive score for HCC diagnosis. The characteristics of different diagnostic tests for HCC diagnosis were investigated by the means of receiver operating characteristic (ROC) curves and compared using the empirical non-inferiority test of the AUROCs. A *p*-value of <0.05 was considered for statistical significance. Statistical analysis was conducted using NCSS 9 and MedCalc 19.0.7 statistical software packages.

## Results

### Patient’s Characteristics

Forty-eight patients with HCC, 38 with LC, and 20 healthy volunteers were included in this study. The therapeutic procedure in the HCC study group was liver resection in 42 patients (87.5%) and liver transplantation in 6 patients (12.5%). The following patient’s clinical characteristics were registered: age, gender, etiology of liver disease, liver function parameters (INR, total bilirubin level, serum creatinine, serum albumin), MELD score, Child Pugh class, the presence of ascites, and baseline AFP level (ng/mL). Patient’s characteristics are depicted in Table 1. HCV etiology was the most prevalent in our patients. There were significantly older patients in the HCC group, whereas liver function parameters were more frequently abnormal in the LC group, in comparison to the HCC study group, explained by the fact that, in the HCC study group, liver resection was conducted more frequently than liver transplantation.

**TABLE 1:** Patient’s demographic and clinical characteristics in the two study groups depicted as percentages of respective categories or mean ± standard deviation (LC and HCC).

**Variable**	**LC**	**HCC**	***p*-Value**
Age > 60 years	36.84%	68.75%	0.003
Male	55.26%	56.25%	0.92
HVC etiology	38.89%	52.08%	0.23
HBV etiology	33.33%	33.33%	1
HDV etiology	25%	8.33%	0.07
INR	1.5 ± 0.49	1.12 ± 0.18	<0.0001
Tbil (mg/dl)	2.99 ± 3.13	0.83 ± 0.58	<0.0001
Serum creatinine (mg/dl)	0.88 ± 0.24	0.97 ± 0.31	0.29
Serum albumin (g/dl)	3.34 ± 0.79	3.67 ± 0.72	0.06
MELD score	14.02 ± 5.09	8.83 ± 2.83	<0.0001
Ascites	31.11%	6.25%	0.001
Child Pugh class A	52.63%	91.67%	
Child Pugh class B	28.95%	8.33%	
Child Pugh class C	18.42%	0%	0.0001
Serum AFP (ng/ml)	10.52 ± 18.15	200.55 ± 380.29	0.001
miR-21-5p relative expression	15.17 ± 19.29	47.96 ± 71.31	0.017
miR-92a-3p relative expression	1.24 ± 1.18	0.61 ± 0.99	0.0005

*HBV, hepatitis B virus; HCV, hepatitis C virus; HDV, hepatitis D virus; INR, international normalized ratio; Tbil, total bilirubin; MELD score, model for end-stage liver disease score; AFP, alpha-fetoprotein.*

### Characterization of Plasma-Derived Exosomes

Exosome isolation and characterization are still major scientific challenges (Lotvall et al., 2014), thus optimizing techniques to isolate exosomes for clinical applications is essential for further biomarker studies.

To validate the exosome isolation methods, the characterization study was performed on samples of a small group of eight patients (four HCC, two LC, two C). The analysis has shown that the exosome population isolated with the commercial TEIp kit shows the same size ranging, morphological, and protein expression aspects compared to the traditional technique UC. The results of this comparison are reported below.

### Nanoparticle Tracking Analysis

**FIGURE 1:**
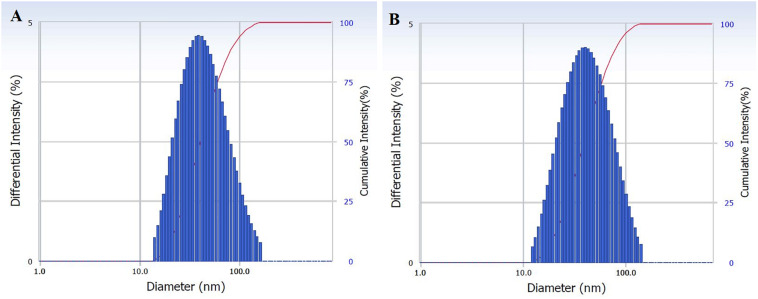
Characterization of isolated exosomes with both methods **(A)** UC and **(B)** TEIp by size distribution of their hydrodynamic diameter using Delsa Nano Analyzer (DelsaNano, Beckman Coulter, Brea, CA, United States). The histograms display the intensity (weighted) size distributions, and the scale of each peak is proportional to the percentage (% amount of the total scattered intensity due to exosome particles).

The size distribution of hydrodynamic diameters of exosomes was determined in real-time using a DelsaNano C particle analyzer. The average size of exosomes isolated by UC was 62.9 ± 45.6 nm (*n* = 4) and for exosomes isolated with TEIp was 66.7 ± 45.3 nm (*n* = 5) (mean ± SD). The overall average size of small EVs varies between 40 and 150 nm; thus, we can observe that the vesicles isolated with both methods have a similar size range (Figures 1A,B).

Exosomes were visualized using two electron microscopy (EM) techniques to compare the outcome of the two different EV isolation methods from the blood of HCC patients (TEIp and UC) and validate the presence of EVs in samples for further analysis.

**FIGURE 2:**
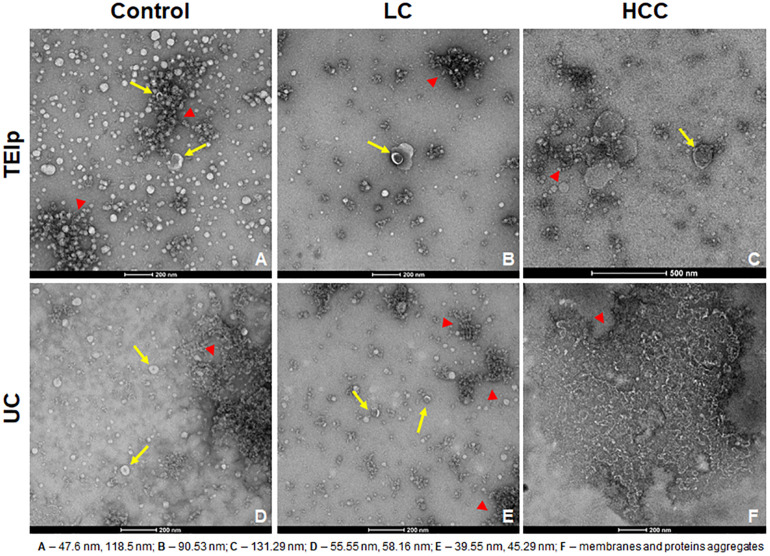
Gallery of negative stain digital micrographs showing cup-shaped exosomes with size ranging in between 40 and 150 nm (yellow arrows) and lipoprotein contamination (red triangles) in samples isolated from patients plasma by TEIp kit **(A–C)** or UC **(D–F)**.

Negative staining of the exosome samples isolated with each of the two methods showed cup-shaped particles within the anticipated size range for exosomes. Moreover, we observed that exosomes isolated with the TEIp kit have a more uniform distribution and a much higher density per grid as compared to the samples obtained by UC at 120,000 × *g* (Figure 2). Even though both types of samples had visible lipid and protein contamination, the EVs isolated by TEIp kit showed significantly reduced levels of membrane and protein aggregates, which were abundant in UC samples (Figure 2F).

In addition to negative stain, cryoTEM yielded additional information on exosomes regarding their structure, membrane, and lumen since lipid bilayers and vesicle internal structure could be visualized (Simons and Raposo, 2009).

### Transmission Electron Cryomicroscopy

**FIGURE 3:**
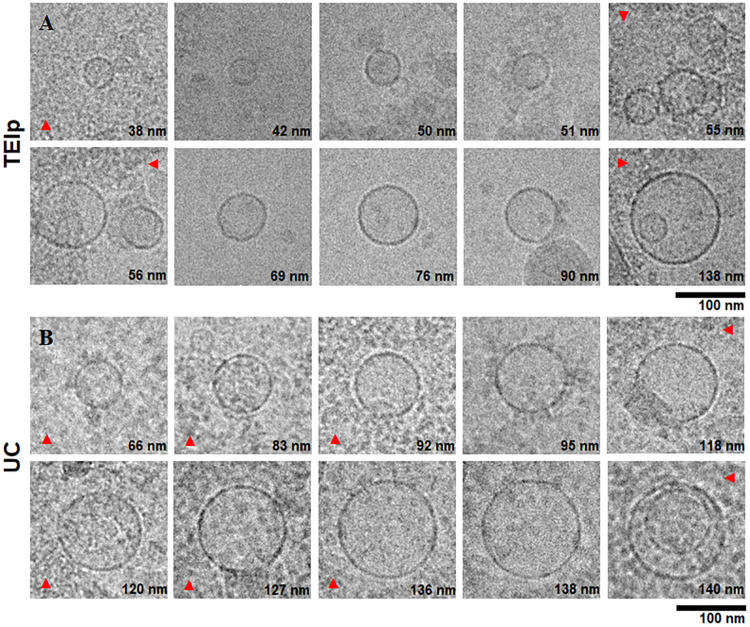
Characterization of small extracellular vesicles by cryo-electron microscopy. Gallery of digital micrographs showing small EVs and lipoprotein contamination (red arrows) in samples isolated from LC patients plasma by exosome isolation TEIp kit **(A)** (top panel) and UC **(B)** (bottom panel).

The morphological analysis of small EVs from plasma revealed by cryoTEM showed clearly defined round structures with a visible lipid bilayer, thus confirming the efficiency of isolation of a heterogenous population of EVs, ranging in size between 40 and 150 nm (Figure 3). For all samples isolated with both methods, we applied a 10× dilution and a short digestion with proteinase K for 10 min at 37°C, and we observed a higher lipoprotein contamination (Figure 3B) and a lower number of vesicles per grid square for samples isolated by ultracentrifugation. To the contrary, TEM showed a far lower concentration of contaminants for samples obtained using the TEIp kit (Figure 3A). As compared to negative stain, cryoTEM is a more powerful tool for differentiating membrane-derived vesicles from other lipid contaminants, stemming from substantial advances in contrast enhancement, detector technology and data processing. Because high levels of contaminants may interfere with downstream applications, cryoTEM is essential for sample screening in EV studies. CryoTEM analysis revealed that both techniques successfully isolated exosomes within the expected size range and morphology, consistent with Delsa Nano particle Analyzer results.

### Western Blotting Analysis

**FIGURE 4:**
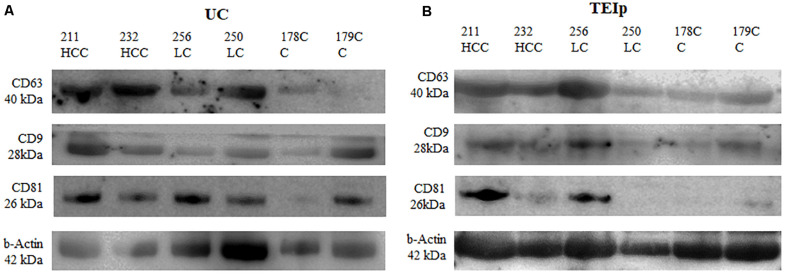
Positive detection for specific exosomal surface markers (CD 63, CD9, and CD81) and for the control-positive marker (b-actin) by immunoblotting. **(A)** exosomes isolated by UC and **(B)** TEIp kit.

The presence of exosomes was validated by immunoblotting of specific exosomal protein markers (CD63, CD81, and CD9). Western blot was performed to compare exosome extraction methods through exosome-specific protein analysis with all three different types of antibodies (CD9, CD81, and CD63). As shown in Figure 4, there was a fairly similar expression for CD63 for both isolation methods; however, the bands in the CD9 protein were less intense after ultracentrifugation than the corresponding sample bands isolated with the TEIp kit. Only the CD81 protein yield showed better results after UC, its expression showing to be strong and uniform across samples with liver pathology compared with controls. The analysis showed that all three exosome-specific proteins are present in the lysate obtained with the TEIp kit. However, looking at pre-equilibrated samples in terms of total amount of protein, we observed less accentuated bands in kit preparations compared with UC. This suggests that there are more non-exosomal proteins in kit preparations that in UC, probably due to precipitation of various other soluble proteins.

**FIGURE 5:**
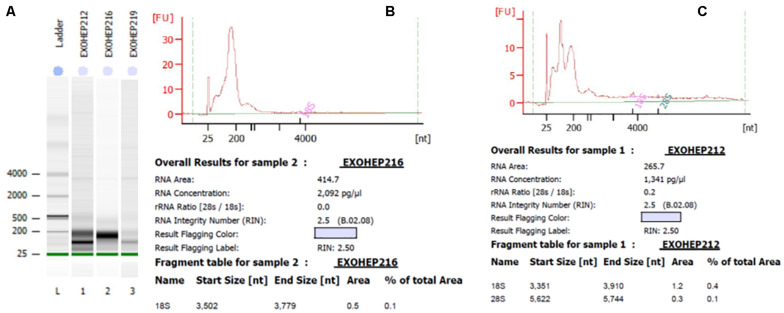
Bioanalyzer analysis of total exosomal RNA by Agilent RNA Pico Chip. The RNA 6000 ladder standard **(A)** first lane contains five RNA fragments between 0.2 and 6 kB. In bands of exosomal RNA from plasma, almost all of the samples revealed a visible band in the small RNA area. **(B,C)** Elecrophoregram for total exosomal RNA.

### The Expression Level of the miR-21-5p and miR-92a-3p in Exosomes Isolated From HCC and Cirrhotic Patients

**FIGURE 6:**
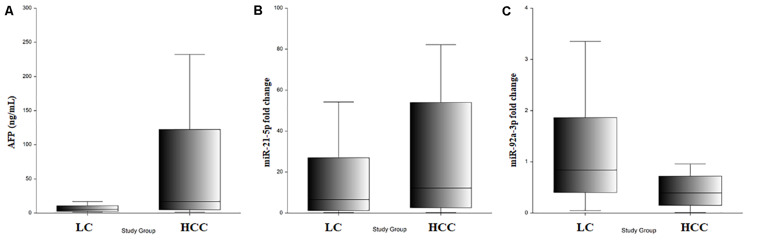
**(A)** Serum AFP level in LC and HCC. **(B)** Exosomal miR-21-5p relative expression in LC and HCC. **(C)** Exosomal miR-92a-3p relative expression in LC and HCC.

Bioanalyzer analysis has shown that exosomal RNA is enriched in small RNAs fraction (Figures 5A–C), thus it could be used to quantify the expression levels of microRNA species. As expected, there was a statistically significant higher level of baseline AFP in the HCC group (*p* = 0.001; Figure 6A). The expression level of the exosomal miR-21-5p was significantly higher in HCC patients in comparison to the LC group (*p* = 0.017; Figure 6B), whereas the expression of miR-92a-3p in exosomes was significantly lower in patients with HCC (*p* = 0.0005; Figure 6C).

Relative expression levels of miR-21-5p and miR-92a-3p were compared considering different patient characteristics defining demographic features and liver disease severity parameters. There were no statistically significant changes in relative expression identified (Table 2A). Furthermore, relative expression levels of miR-21-5p and miR-92a-3p were compared between subgroups defined by significant tumor characteristics: number of tumors, largest tumor diameter, Milan criteria, baseline AFP level with two cutoffs (20 and 100 ng/ml), and no statistically significant differences were noted either (Table 2B).

**TABLE 2:** Relative gene expression (fold change in gene expression) of miR-21-5p and miR-92a-3p according to different patient’s clinical and tumoral characteristics.

**Variable**	**miR-92a-3p**	***p*-Value**	**miR-21-5p**	***p*-Value**
**A: miR relative expression levels according to clinical characteristics**				
**(HCC and LC cases)**				
Age > 60 years	1.06 ± 1.37		37.12 ± 68.17	
Age < 60 years	0.69 ± 0.67	0.4	29.07 ± 39.86	0.71
Male	0.85 ± 1.07		23.36 ± 34.92	
Female	0.93 ± 1.19	0.74	46.25 ± 74.82	0.64
VHC etiology	0.89 ± 1.32		29.53 ± 52.51	
Non-HCV etiology	0.88 ± 0.91	0.96	36.11 ± 61.66	0.33
HBV etiology	0.69 ± 0.75		38.04 ± 69.54	
Non-HBV etiology	0.98 ± 1.26	0.5	30.56 ± 50.7	0.36
HDV etiology	0.86 ± 0.83		28.6 ± 38.91	
Non-HDV etiology	0.89 ± 1.16	0.34	33.87 ± 60.30	0.68
Abnormal INR (>1.27)	1.15 ± 1.1		20.56 ± 32.47	
Normal INR	0.75 ± 1.11	0.11	40.39 ± 65.69	0.18
Low albumin (<3.5 g/dl)	0.92 ± 0.95		29.29 ± 40.46	
Normal albumin	0.86 ± 1.25	0.12	37.28 ± 68.87	0.86
Abnormal Tbil (>1.2 mg/dl)	1.07 ± 1.1		24.38 ± 59.67	
Normal Tbil	0.79 ± 1.12	0.13	38.34 ± 55.35	0.16
High creatinine (>1.3 mg/dl)	0.65 ± 0.88		30.14 ± 30.79	
Normal creatinine	0.91 ± 1.14	0.43	33.77 ± 58.81	0.98
MELD > 15	1.02 ± 0.93		11.47 ± 14.37	
MELD < 15	0.86 ± 1.15	0.13	37.75 ± 61.07	0.11
Ascites – Yes	0.82 ± 1.02		25.96 ± 39.79	
Ascites – No	0.91 ± 1.16	0.95	35.73 ± 62.79	0.87
**B: miR relative expression levels according to tumoral characteristics**				
**(HCC cases)**				
Single nodule	0.70 ± 1.15		45.35 ± 71.81	
Multiple nodules	0.42 ± 0.30	0.89	57.44 ± 74.30	0.94
Diameter >5 cm	0.63 ± 0.75		45.09 ± 65.48	
Diameter <5 cm	0.62 ± 11.1	0.77	50.55 ± 75.97	1
Outside Milan criteria	0.62 ± 0.74		39.98 ± 63.74	
Within Milan criteria	0.62 ± 1.13	0.77	53.63 ± 76.73	0.47
AFP > 20 ng/ml	0.71 ± 1.26		52.51 ± 85.51	
AFP < 20 ng/ml	0.52 ± 0.65	0.45	43.77 ± 56.73	0.53
AFP > 100 ng/ml	0.92 ± 1.59		34.36 ± 58.05	
AFP < 100 ng/ml	0.49 ± 0.58	0.2	53.56 ± 76.18	0.3

The AUROC for HCC diagnosis for serum AFP in our study group was 0.72.

Using logistic regression, a statistical model to predict HCC diagnosis including baseline serum AFP and relative expression levels of miR-21-5p and miR-92a-3p was generated. Based on the new prediction model, the probability of HCC diagnosis could be calculated as follows: Prob (HCC) = 1/[1 + Exp(−XB)], where XB = −0.26−1.30 × RQ_ miR-92a-3p + 0.020 × Serum_AFP + 0.025 × RQ_ miR-21-5p.

**FIGURE 7:**
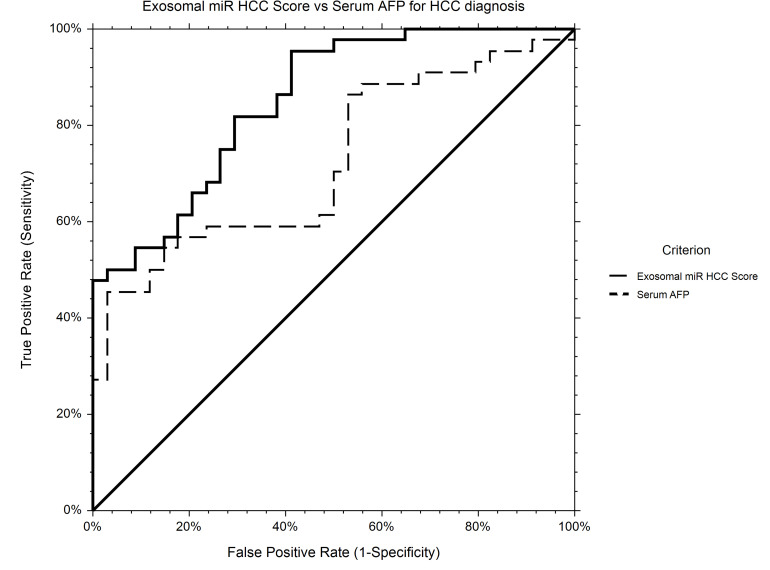
ROC curve for HCC diagnosis for *Exosomal miR HCC Score* in comparison to *Serum AFP*. *Exosomal miR HCC score* (AUROC 0.85) is calculated based on a logistic regression equation including exosomal relative expression of miR-21-5p, miR-92a-3p, and serum AFP level and is significantly better for HCC diagnosis in comparison to AFP alone (AUROC 0.72).

The AUROC of the new score for HCC diagnosis is 0.85, performing significantly better that AFP alone (*p* = 0.0007) as an HCC screening tool (Figure 7).

## Discussion

In clinical practice, the most widely used serological markers for HCC screening are serum AFP, Des-gamma carboxyprothrombin (DCP), and human protein-induced vitamin K absence (PIVKA II) (Galle et al., 2018). According to the most recent EASL guidelines, the cutoff value of 20 ng/ml for AFP shows good sensitivity for HCC diagnosis while the conventional AFP cutoff value of 200 ng/ml is associated with satisfactory specificity and positive predictive value (i.e., low false positivity) of >97% but at a cost of lower sensitivity and negative predictive value (Galle et al., 2018). Early diagnosis of HCC represents a challenge due to lack of reliable biomarkers, emphasizing the need for new diagnostic tools. Over the past years, the new diagnostic concept known as “liquid biopsy” has emerged, and it is also relevant for HCC screening. Circulating tumor cells and circulating tumor DNA are most frequently investigated as liquid biopsy. Exosomes or tumor-educated platelets comprising miRNAs and extracellular RNA are also considered liquid biopsy specimens (Ye et al., 2019). Liquid biopsy has several advantages: non-invasiveness, dynamic monitoring, and the most important of all, overcoming the limit of spatial and temporal tumor heterogeneity. Exosome populations, however, are notoriously heterogeneous and hard to distinguish from spherical lipoproteins when using conventional methods. Therefore, validating the presence of these small EVs in patient samples through imaging methods, prior to other downstream applications, is crucial. In our study, EVs were isolated from plasma samples by precipitation with a reagent based on polyethylene glycol. The presence of EVs in patient’s samples was validated using two EM techniques, cryo-electron microscopy and negative stain, and further characterized based on morphological aspects; diameter and molecular weight; CD63, CD9, and CD81 protein markers; and exosomal quality and quantity. The isolation of EVs using a commercially available kit facilitates the workflow in a translational medicine approach, simplifying the diagnostic procedure for clinical practice.

The present work represents a validation trial of two miRNA candidates selected from an miRNA panel obtained following a comprehensive transcriptomic analysis of tissue, serum, and serum exosomes from HCC patients in a previous study by our group (Mjelle et al., 2019). Briefly, paired tissue, serum, and serum exosome sequencing has indicated a correlation of miR-21 between serum exosomes and tumor tissue, supporting the notion that miR-21 could be exported from tissue to circulation via exosomes. On the contrary, tumoral miR-92a-3p was under-expressed, suggesting that it could be less encountered in exosomal fractions. Taken into account their differential expression in our previous work, the aim of the present study was to investigate the diagnostic utility of these two exosomal miRNAs (miR-21-5p and miR-92a-3p) for HCC diagnosis during a screening program. The present study recruited a different study population directly from the daily clinical practice. All the patients included in present study were subject to the HCC screening protocol that led either to the detection of HCC and a subsequent curative procedure (liver resection or liver transplantation) or were still under the HCC screening protocol while on the waiting list for liver transplantation.

MicroRNAs have been proposed for HCC diagnosis, but most studies are focused on individual miRNAs (Ji et al., 2018). There are, however, some emerging models including multiple miRNAs for HCC diagnosis reported in the literature. The study by Motawi et al. (2015) reveals a serum miRNA panel of miR-19a, miR-146a, miR-195, and miR-192 with high diagnostic accuracy for HCC diagnosis in the context of HCV infection. It has been shown that differentially expressed serum miRNAs, integrated in a 2-miRNA panel according to the regression equation *p* = −2.988 + 1.299 × miR-27b-3p + 1.245 × miR-192-5p are relevant for HBV-related HCC diagnosis (Zhu et al., 2017).

Other recent studies based on bioinformatics analysis of available data sets show that miRNA panels, including miR-221 and miR-99c, are more effective in HCC diagnosis than the traditional serum marker AFP. This combination has a high diagnostic accuracy with AUROC > 0.9 (Ji et al., 2018). In another study conducted by Zhang et al. (2017), a panel consisting of three serum miRNAs (miR-92-3p, miR-107, and miR-3126-5p) and AFP was significantly better for discriminating the early stage HCC patients and low-level AFP HCC patients from controls (Zhang et al., 2017).

Exosomal miRNAs expression may be used as a serum biomarker instead of serum miRNA expression. Wang et al. (2018) show that serum exosomal levels of miR-122, miR-148a, and miR-1246 are significantly higher in HCC than in LC and normal control subjects. Furthermore, there were no differences in miRNAs expression among studied groups taking into account other variables associated with reduced hepatic synthetic function during liver disease progression (albumin and prothrombin time). This is concordant with our results, showing no difference in miR-21-5p and miR-92a-3p expression according to liver function parameters alone or as defined by MELD score or Child-Pugh class. This finding is significant since it facilitates the use of these miRNA biomarkers irrespective of liver disease severity, during screening and surveillance protocols for HCC.

Liao et al. (2015) show that miRNA-21 is expressed in tumor tissue and derived exosomes. Similarly, we have previously shown a consistent positive correlation of miR-21 between serum exosomes and tumor tissue, indicating that miR-21 could be exported from tissue to circulation via exosomes (Mjelle et al., 2019). Moreover, comparing serum and exosomal levels of the miR-21 in patients with HCC, chronic hepatitis, and healthy controls, Wang et al. (2014) suggest that exosomes are the main vehicle of the miRNAs. In this study, miR-21 expression was significantly higher in exosomes compared to exosome-depleted supernatants and whole serum (Wang et al., 2014). Pu et al. (2018) show that the ratio of miR-144-3p/miR-21-5p investigated in EVs extracted from two groups of patients (HCC and chronic hepatitis B) was significantly higher than in EV-depleted serum. Furthermore, statistical analysis has indicated that the miR-144-3p/miR-21-5p ratio performs better than serum AFP for HCC diagnosis (area under the ROC curve 0.78) (Pu et al., 2018). Our research aimed to demonstrate whether serum exosomal miR-21-5p and miR-92a-3p expression level could distinguish patients with HCC from liver cirrhotic patients as an integrated biomarker panel, irrespective of liver disease etiology.

Fornari et al. (2015) show that HCC-derived exosomes carry miR-519d, miR-21, miR-221, and miR-1228 and that circulating and tissue expression levels are correlated. The authors suggest that miR-519d performs better than AFP for HCC diagnosis. On the contrary, miR-21 and miR-221 had a more heterogenous expression in different populations, correlated to viral prevalence and ethnicity (Fornari et al., 2015). This is concordant to our study, which did not show better diagnostic accuracy for miR-21-5p alone in comparison to AFP for diagnosis of HCC (AUROC 0.65 – data not shown).

In other studies, however, serum miR-21 proved to be an independent predictor for tumor recurrence following treatment, stronger than AFP (Tomimaru et al., 2012), raising important physiopathological questions.

Zhou et al. (2018) show that exosomal miR-21 derived from HCC cell lines is increased and promotes cancer progression by activating cancer-associated fibroblasts (CAFs). MiR-21 can be involved in transformation of normal hepatic stellate cells (HSCs) into CAFs by directly targeting the phosphatase and tensin homolog (PTEN) gene, leading to activation of PDK1/AKT signaling pathway in hHSCs and secretion of angiogenic cytokines, including vascular endothelial growth factor (VEGF), matrix metallopeptidase 2 (MMP-2), matrix metallopeptidase 9 (MMP-9), fibroblast growth factor 2 (FGF2), and transforming growth factor (TGF). Experimental data indicates that high levels of serum exosomal miRNA-21 correlate with activation of CAFs and with higher vessel density in patients with HCC. More importantly, the high expression of miR-21 derived from serum exosomes showed a positive correlation with survival rate in patients with HCC. Furthermore these results suggest that miR-21 may be also a potential target for HCC prevention and treatment (Zhou et al., 2018). Recent studies have shown that overexpression of miR-21 could increase the methylation level of the phosphatase and tensin homolog pseudogene 1 (PTENp1) promoter by regulating ten eleven translocation (TET) expression, thereby inhibiting PTENp1 expression, thus leading to downregulation of PTEN and affecting the growth of HCC cells (Cao et al., 2019). MiR-21 can promote the proliferation and metastasis of HCC cells by inhibiting the PTEN expression (Meng et al., 2007), reversion inducing cysteine-rich protein with kazal motifs (RECK) and human sulphatase-1 (Sulf-1) (Bao et al., 2013) and can induce resistance at chemotherapeutic drugs in HCC cells (He et al., 2015). Thus, differentially expressed exosomal miR-21 is an important biomarker for both early detection of HCC and disease progression.

Data related to miR-92a expression in HCC is still controversial. Several authors emphasize that circulating miR-92a is significantly under-expressed in HCC and that the dysregulated pattern is related to cancer development and HCC progression (Ohyashiki et al., 2010; Wang et al., 2017). Expression level of exosome-derived miR-92a, significantly decreased in plasma of HCC patients in comparison to controls, has also been proposed as a potential diagnostic biomarker for HCC (Ohno et al., 2013; Qu et al., 2015). Our results are in concordance with this finding as we also found an under-expression of miR-92a in plasma exosomes of HCC cases in our study group, in comparison to LC. On the other hand, *in vivo* studies have suggested that the downregulation of miR-92a suppresses the biological processes of tumor growth in HCC and that F-box and WD repeat domain-containing 7 (PBXW7) is a direct target of miR-92a, promoting tumor progression in this setting (Yang et al., 2015). These contradictory results could be explained by several technical aspects, such as miRNA purification/detection protocol, sample storage conditions, and different internal controls and also by the type of analyzed samples: serum or plasma. There is also data suggesting that dysregulation of miR-92a is, in fact, cancer type–specific (Ohno et al., 2013). Overexpression of miR-92a could induce various carcinogenesis processes in different tumors. It has been shown to promote ovarian cancer cell adhesion, proliferation, and invasion by suppressing integrin α5 expression, contrasting the effects observed in HCC (Ohyagi-Hara et al., 2013).

In our study, the diagnostic accuracy for HCC for exosomal relative expression of miR-21-5p, miR-92a-3p, and serum AFP were analyzed individually and combined. The statistical model integrating the two selected miRNAs and AFP could be proposed as a novel screening tool for HCC as it performed best for differentiating HCC from cirrhotic controls in comparison to the individual variables.

The addition of this miRNA panel to traditional serological tumor markers may improve the diagnostic accuracy for HCC detection and, in a translational setting, could be more easily and conveniently performed in comparison to other liquid biopsy techniques, isolating circulating tumor cells or circulating tumor DNA. Model validation in a larger cohort of plasma exosomal samples could improve diagnostic accuracy.

## Conclusion

In summary, exosome trafficking proteins, miRNAs, and lncRNAs are important emerging diagnostic biomarkers for HCC. Our exosomal miR-HCC score, integrating exosomal miR-21-5p, miR-92a-3p expression, and serum AFP, could provide a novel diagnostic tool for HCC screening in clinical practice.

## Data Availability Statement

All datasets generated for this study are included in the article/Supplementary Material.

## Ethics Statement

This study, involving human participants, was reviewed and approved by the Ethics Committee of Fundeni Clinical Institute. The patients provided their written informed consent to participate in the study.

## Author Contributions

AS, RI, AD, and SD contributed to the design of the study. AS, DC, MC-E, LC, and TF performed the experiments. AS, RI, SI, DC, and SD organized the study database. RI performed the statistical analysis and clinical results interpretation. AS and DC wrote the first draft of the manuscript. SD, RI, SI, MC-E, and LC wrote sections of the manuscript. IP, SD, NB, LS, LG, and AD supervised the studies and reviewed the manuscript. All authors contributed to manuscript revision, read and approved the submitted version.

## Conflict of Interest

The authors declare that the research was conducted in the absence of any commercial or financial relationships that could be construed as a potential conflict of interest.
