# Supplementary material for: Plasma Small Extracellular Vesicles Derived miR-21-5p and miR-92a-3p as Potential Biomarkers for Hepatocellular Carcinoma Screening
Source: Front Genet. 2020 Jul 23;11:712. doi: 10.3389/fgene.2020.00712 (PMC7391066; doi:10.3389/fgene.2020.00712)
Supplement: Supplementary file 1 [file Table_1.DOCX]

***S1 – Supplementary data***

***Methods and materials***

*In our study, relative quantification of miRNAs expression in HCC and LC versus healthy controls was calculated using the 2^-ΔΔCt^ method (fold change of the target miRs expression between disease and control) (1, 2). The term ΔΔCt measures the relative change of expression of target gene from disease to control compared to the reference gene (miR-16). For samples included in the study the following calculation was applied:*

***(Eq1) ΔCt = Ct _(target miRs)_ - Ct _(reference miR)_:***

*ΔCt _(HCC samples)_ = AvgCt _(target miR)_ - AvgCt _(miR-16)_*

*ΔCt _(LC samples)_ = AvgCt _(target miR)_ - AvgCt _(miR-16)_*

*ΔCt _(C samples)_ = AvgCt _(target miR)_ - AvgCt _(miR-16)_*

*and*

***(Eq 2) - ΔΔCt _HCCvsC_= - (ΔCt _(HCC samples)_ - ΔCt _(C samples)_),***

***- ΔΔCt _LCvsC_ = - (ΔCt _(LC samples)_ - ΔCt _(C samples)_***)

**Fold change: 2^−ΔΔCt^ = 2 ^- ΔΔCt HCCvsC^ and 2 ^- ΔΔCtLCvsC^**

References:

1. Livak KJ and Schmittgen TD. Analysis of relative gene expression data using real-time quantitative PCR and the 2(-Delta Delta C(T)) method. Methods. 25:402–408. 2001

2. Schmittgen TD, Livak KJ. Analyzing real-time PCR data by the comparative C(T) method. Nat Protoc. 2008;3(6):1101-8
